# Supplementary material for: An information-theoretic framework for deciphering pleiotropic and noisy biochemical signaling
Source: Nat Commun. 2018 Nov 2;9:4591. doi: 10.1038/s41467-018-07085-1 (PMC6214929; doi:10.1038/s41467-018-07085-1)
Supplement: Supplementary file 2 — Supplementary Information [file 41467_2018_7085_MOESM1_ESM.pdf]

Supplementary Information

An information-theoretic framework for deciphering pleiotropic and noisy biochemical signaling

Jetka at al.

Supplementary Methods

1 Derivations

1.1 Background

We consider a general model of a biochemical reaction network represented as a conditional probability distribution, $P(Y|X=x)$, of an output value, Y, given an input value, x. Both input and output can in principle be multidimensional, i.e. $Y=(Y_1,...,Y_l)$ and $x=(x_1,...,x_k)$. The information capacity, C^*, is then defined as the maximal mutual information, $I(X,Y)$, between the input, X, and the output, Y, over a set of continuous and strictly positive probability distributions. Precisely,

$$C^* = \max_{P(x)} I(X,Y) = \max_{P(x)} \int_{\mathcal{X}} \int_{\mathcal{Y}} P(x)P(y|X=x) \log_2 \frac{P(y|X=x)}{P(y)} dydx, \qquad \text{(S.1)}$$

where $\mathcal{X}$ and $\mathcal{Y}$ denote spaces of possible values of the input and output, respectively. The distribution, for which the maximum is achieved is referred to as the optimal input distribution and is denoted as, $P^*(x)$. Calculation of the information capacity, C^*, and finding the optimal input distribution, $P^*(x)$, is commonly solved by Blahut-Arimoto algorithm (BAA) [S1, S2], which enforces discretisation of the input and output values. Upon discretisation, the maximum of the Eq. S.1 can be found using several conventional optimisation schemes, e.g. steepest decent method [S3, S4]. Whereas BAA is applied across different disciplines [S5, S6, S7], in the context of biochemical networks, an attractive and computationally undemanding approach of 'small noise approximation' (SN) was proposed in [S3]. This however assumes limited stochasticity of the examined system. We currently lack examples where the information capacity is calculated for biochemical signaling pathways with multiple inputs and multiple outputs, as the existing methods either lack generality or are computationally prohibitive. To overcome these limitations, we take an asymptotic approach. Specifically, we consider N independent copies of the same signaling system

$$Y^{(j)} \sim P(Y|X=x), \qquad \text{(S.2)}$$

for j going from 1 to N, and their joint output

$$Y_N = (Y^{(1)}, \ldots, Y^{(N)}).$$

The communication channel with the input X and the output, Y_N, is then defined as

$$P(Y_N|X=x) = P((Y^{(1)}, \ldots, Y^{(N)})|X=x) = \prod_{j=1}^{N} P(Y^{(j)}|X=x),$$

where the latter equality results from the assumed independence of individual outputs. Then, the corresponding information capacity problem has the form

$$C_N^* = \max_{P(x)} I(X, Y_N) \qquad \text{(S.3)}$$

with the optimal input distribution denoted as $P_N^*(x)$.

Note, that in the above notation C_1^* and C^* are equivalent.

1.2 Asymptotically optimal input distribution, $P_{JP}^*(x)$

The problem of finding the distribution $P_N^*(x)$ has been solved in statistics in the context of non-informative priors. Precisely, it has been shown (e.g. in [S8, S9, S10]) that under general regularity conditions (see the references [S9] and [S11] for details of these conditions)

$$P_N^*(x) \xrightarrow{N\to\infty} P_{JP}^*(x), \qquad \text{(S.4)}$$

where $P^*_{JP}(x)$ is the Jeffreys prior [S12] defined as

$$P^*_{JP}(x) \propto \sqrt{|\mathrm{FIM}(x)|}, \tag{S.5}$$

where $|\cdot|$ is the matrix determinant; FIM is the Fisher information matrix

$$\mathrm{FIM}(x)_{i,j} = \mathbb{E}\left(\frac{\partial \ln(P(Y|X=x))}{\partial x_i}\frac{\partial \ln(P(Y|X=x))}{\partial x_j}\right), \tag{S.6}$$

and $\mathbb{E}(\cdot)$ is the expected value with respect to Y at a fixed x. The exact asymptotic description of the optimal input distribution by the Jeffreys prior, Eq. S.4, is derived without any approximations to the input-output model, $P(Y|X=x)$ [S9].

1.3 Optimal input distribution as a bayesian non-informative prior

For illustration of the relationship between the maximisation of the Eq. S.3 and the problem of finding a non-informative prior distribution in Bayesian inference, consider X to be a parameter vector inferred from data Y_N. In Bayesian statistics the prior distribution $P(X)$ is translated into a posterior distribution, $P(X|Y_N = y_n)$, representing the knowledge about the parameter X present in the observed data y_N. If, in the inference process, no prior knowledge about the parameter, X, is available, it is desirable to use a non-informative prior distribution, i.e. a prior that impacts the results of estimation as little as possible. Non-informativeness of a prior distribution can be expressed in terms of the mutual information. Consider the mutual information, $I(X, Y_N)$, written in terms of entropy differences, Eq. 14-17 of the main paper,

$$I(X, Y_N) = H(X) - H(X|Y_N), \tag{S.7}$$

where $H(X)$ is the entropy of the prior distribution $P(x)$ and $H(X|Y_N)$ is the average entropy of the posterior distribution, $P(X|Y_N = y_n)$. The entropy $H(X)$ represents the overall prior uncertainty about the parameter, whereas $H(X|Y_N)$ quantifies the average uncertainty about the parameter, when data y_N is available. Therefore, the mutual information $I(X, Y_N)$ can be interpreted as a measure of how much can be learned from data Y_N, i.e. the missing knowledge. Therefore, among all considered priors the one that maximises the missing knowledge, quantified by the mutual information $I(X, Y_N)$, can be seen as the least informative. A prior distribution that is least informative in terms of the mutual information between X and Y_N is called the reference prior. In the asymptotic scenario, i.e. large N, under certain regularity conditions [S9, S11] the Jeffreys prior (S.5) is asymptotically least informative [S8, S9, S10, S13, S14].

1.4 Derivation of C^*_A

A strict and elegant proof that

$$C^*_N - \frac{k}{2}\log_2(N) \xrightarrow[N\to\infty]{} C^*_A, \tag{S.8}$$

where

$$C^*_A = \log_2\left(\frac{1}{(2\pi e)^{\frac{k}{2}}}\int_{\mathcal{X}} |FIM(x)|^{\frac{1}{2}} dx\right), \tag{S.9}$$

is shown in [S9]. Here, for the convenience of the reader, largely following [S15], we present a scheme behind one of the possible derivations.

Primarily, instead of the mutual information $I(X, Y_N)$ between the signal, X, and the output, Y_N, consider the mutual information $I(X, \hat{X})$ between the signal, X, and the maximum likelihood estimator

(MLE), $\hat{X}$, of the signal, X. Obviously, $I(X,Y) \geq I(X,\hat{X})$, i.e. the estimator cannot contain more information about the signal than the output. Nevertheless, it can be shown [S16] that for large N

$$I(X,Y_N) \approx I(X,\hat{X})$$

as MLE is 'asymptotically sufficient' [S17]. Therefore, $I(X,\hat{X})$ should serve as a good proxy for $I(X,Y_N)$

$$I(X,Y_N) \approx H(\hat{X}) - H(\hat{X}|X).$$

Secondly, the asymptotic theory of the MLEs states that $\hat{X}$, obtained from N independent copies of Y, has the Gaussian distribution [S16]

$$\hat{X} \sim \mathcal{N}(x, \frac{1}{N} FIM(x)^{-1}).$$

Using the formula for the entropy of k-dimensional normal distribution[1], we get

$$I(X,Y_N) \approx H(\hat{X}) - \int_{\mathcal{X}} P(x) \frac{1}{2} \log_2 \frac{(2\pi e)^k}{|N \cdot FIM(x)|} dx.$$

Thirdly, if the variability of the signal, X, is large compared to uncertainty in the estimator then $H(X) \approx H(\hat{X})$. Hence, we can write

$$I(X,Y_N) \approx H(X) - \int_{\mathcal{X}} P(x) \frac{1}{2} \log_2 \frac{(2\pi e)^k}{N^k \cdot |FIM(x)|} dx. \tag{S.10}$$

Fourthly, Eq. S.3 states that C_N^* is maximised for $P_{JP}^*(x) \propto \sqrt{|FIM(x)|}$, therefore denoting

$$V = \int_{\mathcal{X}} \sqrt{|FIM(x)|} dx$$

and substituting

$$P(x) = \frac{1}{V} \sqrt{|FIM(x)|},$$

into Eq. S.10 yields

$$\begin{aligned}
C_N^* &\approx -\int_{\mathcal{X}} \frac{1}{V} |FIM(x)|^{\frac{1}{2}} \log_2 \left(\frac{1}{V} |FIM(x)|^{\frac{1}{2}} \right) dx - \int_{\mathcal{X}} \frac{1}{V} |FIM(x)|^{\frac{1}{2}} \log_2 \frac{(2\pi e)^{\frac{k}{2}}}{N^{\frac{k}{2}} \cdot |FIM(x)|^{\frac{1}{2}}} dx \\
&= -\int_{\mathcal{X}} \frac{1}{V} |FIM(x)|^{\frac{1}{2}} \log_2 \frac{1}{V} dx - \int_{\mathcal{X}} \frac{1}{V} |FIM(x)|^{\frac{1}{2}} \log_2 |FIM(X)|^{\frac{1}{2}} dx \\
&\quad - \int_{\mathcal{X}} \frac{1}{V} |FIM(x)|^{\frac{1}{2}} \log_2 \left((2\pi e)^{\frac{k}{2}} \right) dx + \int_{\mathcal{X}} \frac{1}{V} |FIM(x)|^{\frac{1}{2}} \log_2 |FIM(x)|^{\frac{1}{2}} dx \\
&\quad + \int_{\mathcal{X}} \frac{1}{V} |FIM(x)|^{\frac{1}{2}} \log_2 N^{\frac{k}{2}} dx \\
&= \log_2 V - \log_2 (2\pi e)^{\frac{k}{2}} + \frac{k}{2} \log_2 N.
\end{aligned}$$

Finally, we obtain

$$C_N^* \approx \frac{k}{2} \log_2 N + \log_2 \left(\frac{1}{(2\pi e)^{\frac{k}{2}}} \int_{\mathcal{X}} |FIM(x)|^{\frac{1}{2}} dx \right). \tag{S.11}$$

[1]If $Z \sim \mathcal{N}(\mu, \Sigma)$ then $H(Z) = \frac{1}{2} \log_2((2\pi e)^k |\Sigma|)$, where $|\cdot|$ is the matrix determinant, and k is the dimension of Z.

1.5 Negative values of asymptotic capacity and an auxiliary approximation C^*_{JP}

As explained in the main paper, the asymptotic information capacity C^*_A can take negative values. This, however, does not result from a caveat of our approach but from mathematical properties of how information scales with increasing N (i.e. the number of receivers / cells). There exist many other widely accepted information theoretic measures that can take negative values. Differential entropy is probably the best-known example. Other examples include interaction information (i.e. certain form of conditional mutual information) or even mutual information in specific settings. (e.g. $H(X) - H(X|Y = y)$ can be negative if $H(X|Y = y)$ is not averaged over all possible y's as in the definition of standard mutual information: $H(X) - H(X|Y)$).

In scenarios, where C^*_A is negative, it is not a good approximation of C^*_1, which is a positive quantity. Therefore, for values of C^*_A close to zero it should be used with caution when aiming to approximate C^*_1. Its asymptotic interpretation is, however, valid also for negative values, as explained in the main paper. Below we describe an alternative approximation that is guaranteed to be positive. To this end, instead of approximating C^*_N with the Eq. S.11, we replace the exact optimal input distribution, $P^*(x)$, in the Eq. S.1 with its asymptotic version, $P^*_{JP}(x)$. We denote this approximation C^*_{JP}. Precisely,

$$C^*_{JP} = \int_{\mathcal{X}} \int_{\mathcal{Y}} P^*_{JP}(x) P(y|X = x) \log_2 \frac{P(y|X = x)}{P(y)} dydx, \tag{S.12}$$

where $P(y|X = x) = \int_{\mathcal{X}} P(y|X = x) P^*_{JP}(x)dx$. In other words, C^*_{JP} is defined as the mutual information between the output, Y, and input, X, being distributed according to the asymptotically optimal input distribution, $P^*_{JP}(x)$. Therefore, similarly to the mutual information, C^*_{JP} is the positive quantity.

In practical applications, calculation of C^*_{JP} is computationally more demanding than of C^*_A. Precisely, calculation of both C^*_A and C^*_{JP} involves evaluation of the Fisher information as wells as requires integration with respect to input values. In addition to the above two steps, computation of C^*_{JP} involves integration with respect to the output for each value of the input. Hence, the difference in the computational cost increases significantly with the dimension of the output, Y, and of the input, X. Moreover, for many distributions, including the exponential family, the Fisher information has an explicit form or can be expressed in terms of derivatives of moments (Section 6). In such scenarios, Fisher information can be computed without evaluating $P(Y|X = x)$. Therefore, computation of C^*_A is in general significantly simpler and computationally less demanding than of C^*_{JP}. Moreover, in contrast to C^*_A, C^*_{JP} cannot be expressed as a relatively simple and conveniently applicable equation that links sensing precision, i.e., Fisher information, with the overall signaling fidelity, quantified as the information capacity.

2 Test model

For comprehensive comparison of the capacities, C^*_A, C^*_{JP}, C^*_{SN} with C^*_1 we have designed a test model. For the test model all methods are computationally feasible. Precisely, we considered a system composed of L identical copies of a biochemical sensor, e.g. a receptor or a kinase. Suppose, each copy can be either in an active or inactive conformation and assume that the probability of each copy being in the active conformation is given as the Michaelis-Menten function of a signal S

$$h(S) = \frac{S/K_\mathrm{d}}{1 + S/K_\mathrm{d}},$$

whereby K_d is the sensor's dissociation constant. In addition, we assumed that the sensors are exposed to competing ligands that resemble the cognate ligand so that the signal S is composed of two sources.

Precisely, $S = X + \frac{1}{\lambda}X_F$, where X and X_F are the concentrations of the cognate (true) and non-cognate (false) ligands, respectively. The sensor's selectivity factor is given by $\lambda = \frac{K_\mathrm{d}^\mathrm{F}}{K_\mathrm{d}}$, where K_d^F is the sensor's dissociation constant for the non-cognate ligand. The higher λ is, the less likely is the sensor to bind the non-cognate ligand. The variability in the non-cognate ligand concentration is represented by the probability distribution $P(X_F)$. Given that each copy takes its conformation independently, the distribution of the number of active sensors, Y, is given by the binomial distribution

$$P(Y|S) = \mathrm{Bin}(h(S), L).$$

Assuming that the concentration of the cognate ligand, X, and the number of active sensors, Y, are the input and output of the system, respectively, then

$$P(Y|X = x) = \int_{\mathcal{X}_F} P\left(Y|S = x + \frac{1}{\lambda}x_F\right) P(X_F = x_F)dx_F, \tag{S.13}$$

represents the input-output relationship of the system with $\mathcal{X}_F$ being the space of possible concentrations of the non-cognate ligands. Changing settings of this model allowed us to thoroughly challenge the tested methods. In total, we have considered 27 different scenarios by combining different variants of the probability distributions $P(X_\mathrm{F})$; sensor copy number, L; and of the selectivity factor, λ. In each scenario, we have calculated capacities as a function of the standard deviation of the non-cognate ligand, σ_{X_F}.

2.1 Test scenarios

To compare the capacities, $C_A^*, C_\mathrm{JP}^*, C_\mathrm{SN}^*, C^*$, we have considered

1. three distributions of the concentrations of the non-cognate ligands, $P(X_F)$: log-normal, exponential and gamma distribution;
2. three values of the copy number: $L \in \{50, 100, 500\}$;
3. three values of the selectivity factor: $\lambda \in \{10, 2, 1\}$;

which in combination gave 27 different scenarios. In all scenarios, we assumed $K_\mathrm{d} = 1$. In each scenario, we have calculated the information capacities as a function of the standard deviation, σ_{X_F}, of the distribution $P(X_F)$. For the log-normal and gamma distributions, we assumed that their mode is equal to 1 to match the sensor's K_d, and adjusted the distribution's mean and variance accordingly. In the case of the exponential distribution the mode cannot be tuned as it is equal to 0 regardless of parameters.

2.2 Numerical comparison

The comparison of the four capacities, $C_A^*, C_\mathrm{JP}^*, C_\mathrm{SN}^*$, and C^*, in all 27 considered scenarios is presented in Supplementary Figure 1 and Supplementary Figure 2. The summary plot resulting from this comparison constitutes Fig. 1 of the main paper.

As discussed in the main paper, C_A^* is on average more accurate than C_SN^*. The more detailed comparison presented here includes C_{JP}^*. It reveals an interesting aspect regarding whether C_{JP}^* or C_A^* is a more accurate approximation of C^*.
In the derivation of C_{JP}^* only one approximation is used, i.e. of the optimal input distribution, Eq. S.12. On the other hand, in the derivation of C_A^*, two approximations are used, i.e. of the information

capacity formula, Eq. S.10, and of the optimal input distribution, the derivation prior to Eq. S.11. The above suggests, that C^*_{JP} should be, in general, a better approximation of C^*. Supplementary Figure 1 and Supplementary Figure 2 show that typically this is the case. However, in certain scenarios, e.g., exponential distribution with $\lambda = 1$ and $L = 500$, C^*_A, is a better approximation than C^*_{JP}, which may seem counter-intuitive. To explain why the above is possible, the following argument should be made. Approximations can either over- or underestimate a true value. If within one approximation, two approximations are used concurrently, each approximation may happen to have an opposite direction. If this is the case, the inaccuracies of two approximation will partly cancel out. Therefore, the seemingly less accurate approximation, C^*_A, which involves two approximations, can have, in certain scenarios, higher accuracy than C^*_{JP}, which involves one approximation.

For twelve representative scenarios, we have also plotted the optimal input distributions calculated using the Blahut - Arimoto algorithm, $P^*(x)$; Jeffreys prior, $P^*_{\mathrm{JP}}(x)$; and small noise approximation $P^*_{\mathrm{SN}}(x)$.Supplementary Figure 3 shows that the calculated distributions exhibit only minor differences.

2.3 Technical details

Fisher information of the model, i.e. of the probability distribution, Eq. S.13, was calculated by numerical integration of the derivative of log-likelihood according to the Eq. S.6. Calculation of the information capacity, C^*_1, using the Blahut-Arimoto algorithm [S1] was implemented in Matlab. Both codes are available upon request.

2.3.1 Comparing continuous and discrete distributions

In the Supplementary Figure 3 we compare the results of the Blahut-Arimoto algorithm, which operates on discrete inputs, with continuous input distributions of other considered methods. Therefore, in order to present it graphically on comparable scales, discrete optimal input distribution of the BAA has been transformed into a continuous probability distribution. Suppose, the discrete probability distribution $\{p_1, p_2, \ldots, p_m\}$ defined on a set of real numbers $x_1 < x_2 < \ldots < x_m$ is the output of the BAA. For such probability distribution we have defined a corresponding piecewise constant density function, $p(x)$,

$$p(x) = \begin{cases} 0 & \text{for} \quad x < x_1 \\ \frac{p_1}{\frac{1}{2}(x_2 - x_1)} & \text{for} \quad x_1 \leq x < \frac{(x_1+x_2)}{2} \\ \frac{p_2}{\frac{1}{2}(x_3 - x_1)} & \text{for} \quad \frac{(x_1+x_2)}{2} \leq x < \frac{(x_2+x_3)}{2} \\ \cdots & \\ \frac{p_i}{\frac{1}{2}(x_{i+1} - x_{i-1})} & \text{for} \quad \frac{(x_{i-1}+x_i)}{2} \leq x < \frac{(x_i+x_{i+1})}{2} \\ \cdots & \\ \frac{p_n}{\frac{1}{2}(x_n - x_{n-1})} & \text{for} \quad \frac{(x_{n-1}+x_n)}{2} \leq x < x_n \\ 0 & \text{for} \quad x_n \leq x \end{cases} .$$

The above definition 'extends' the values of the discrete probabilities, p_i's, around points, x_i's, by placing points of discontinuity half-way between x_{i-1} and x_i, for i changing from 2 to m, and sets probability value proportionally to the discrete probability, p_i and inversly to the length of the interval.

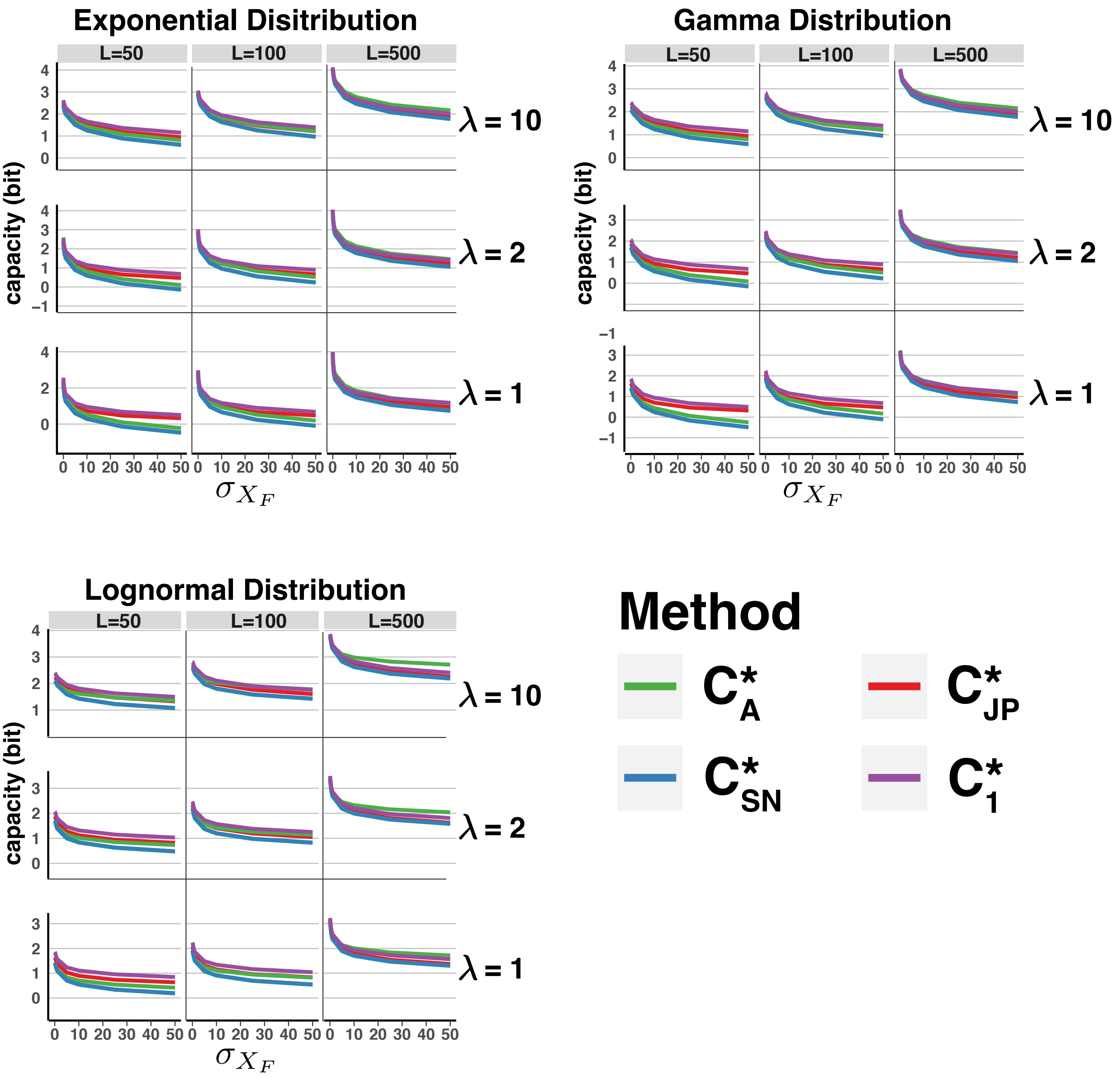

Supplementary Figure 1: Channel capacities $C^*_A, C^*_{\mathrm{JP}}, C^*_{\mathrm{SN}}, C^*_1$ for the biochemical sensor model in 27 considered scenarios.

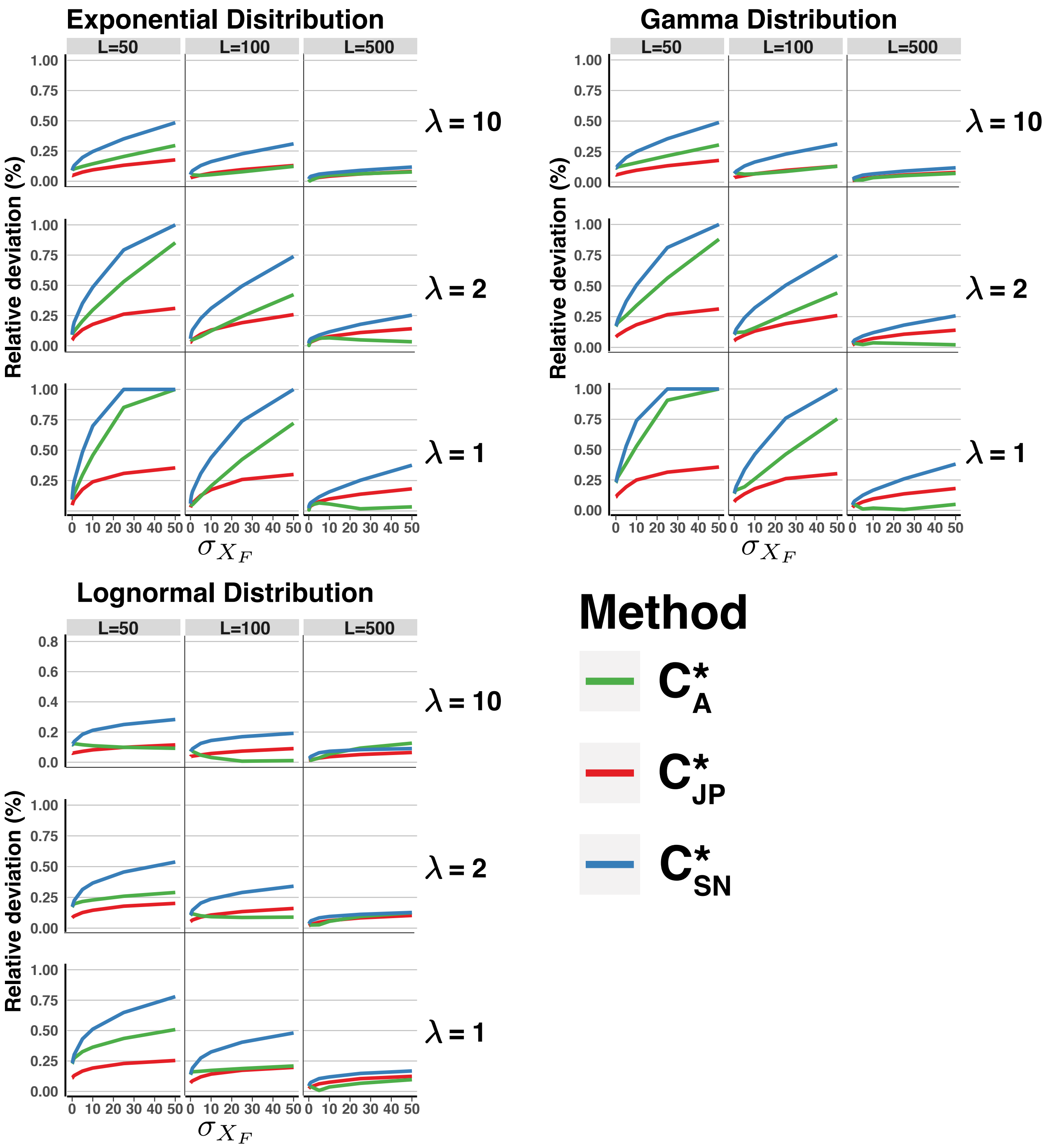

Supplementary Figure 2: Relative deviation of the capacities, C^*_A, C^*_{JP}, and C^*_{SN} with respect to capacity C^*_1 in 27 scenarios of Supplementary Figure 1.

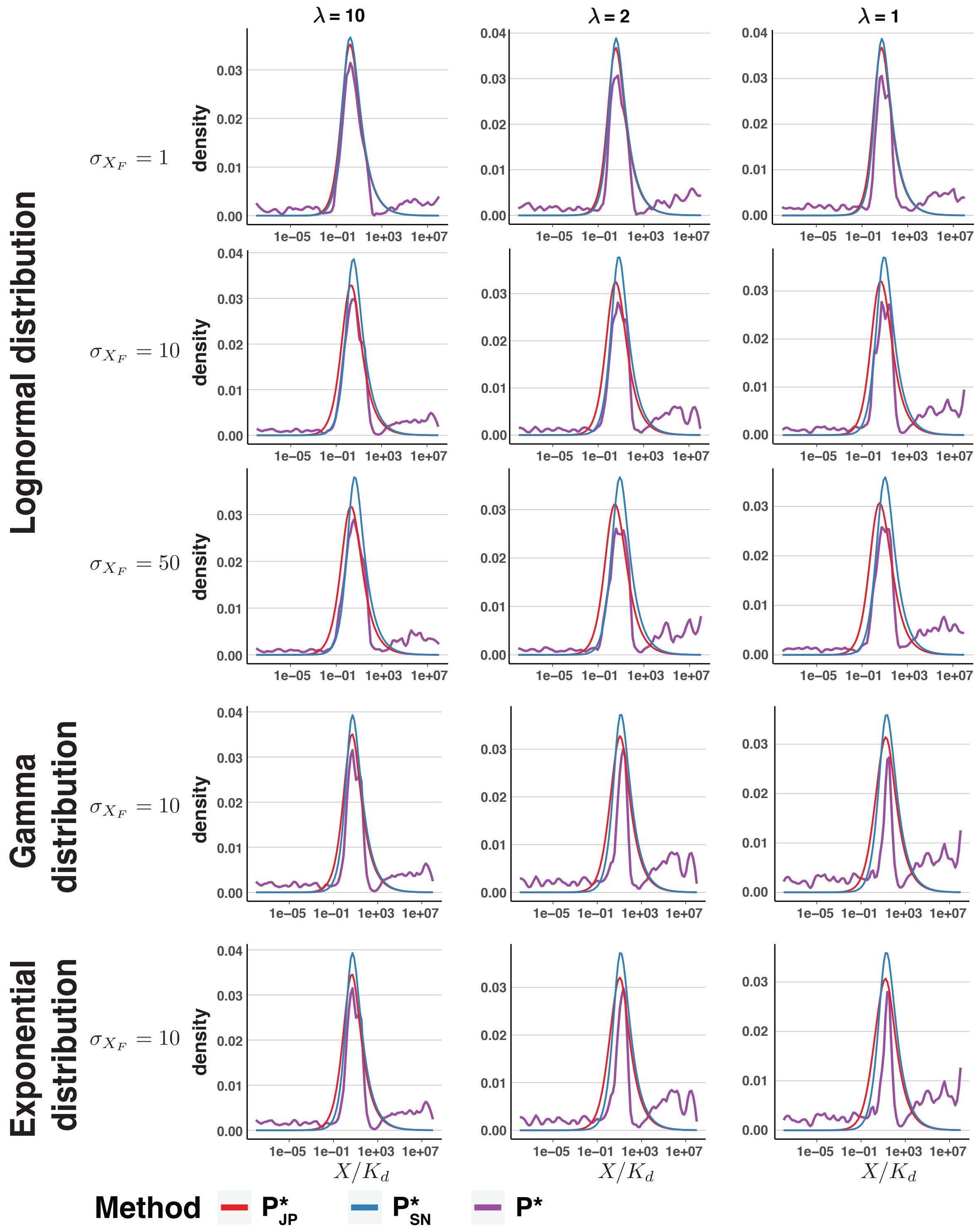

Supplementary Figure 3: Comparison of the optimal input distributions calculated using the Blahut-Arimoto algorithm, $P^*(x)$; Jeffreys prior, $P^*_{\mathrm{JP}}(x)$; and small noise approximation $P^*_{\mathrm{SN}}(x)$, in twelve representative scenarios of the Supplementary Figure 1. For plotting, the discrete distribution of the Blahut-Arimoto algorithm was transformed into continuous distribution as described in Section 2.3.1.

3 Type I and type III interferons signaling model

In humans three different types of interferons (IFNs) exist. Type I IFNs comprise sixteen variants, including IFN-α, whereas type III IFNs consist of four variants, including IFN-$\lambda 1$ [S18, S19, S20]. Type I IFN variants exert their actions through cognate two subunits receptor complexes IFNAR1/IFNAR2. IFNAR1 is constitutively associated with tyrosine kinase 2 (TYK2), whereas IFNAR2 with Janus tyrosine kinase 1 (JAK1). Binding of the type I variant to its cognate receptor complex results in trans-phosphorylation of JAK1 and TYK2. The activated kinases then phosphorylate cytoplasmic tails of the IFNAR1/IFNAR2. Cytoplasmic tails of both receptor complexes serve as docking sites for the recruitment of several proteins, most importantly of STAT1 and STAT2. Following phosphorylation activated STATs form homodimers, p-STAT1/1, or heterodimers, p-STAT1/2, which translocate to the nucleus, where they bind DNA to specific cognate sites in concert with a variety of other nuclear factors and serve as active transcription factors. Dephosphorylation in the nucleus results in nuclear export of STATs and making them available to subsequent phosphorylation/dephosphorylation cycles [S21, S22].

Type III variants, instead of IFNAR1/IFNAR2, bind to the cognate IFNLR1/IL10Rα receptor complex where IFNLR1 is constitutively associated with tyrosine kinase 2 (TYK2), whereas IL10Rα with JAK1 [S23]. Downstream signaling cascade, according to the current understanding [S23, S24, S25], appears to follow the same pattern as described above for type I variants.

As IFN-α and IFN-$\lambda 1$ are often studied in experiments, e.g. [S25, S26], we used these two variants as representatives of the type I and type III IFNs, respectively.

3.1 Model variables

In order to construct the model of type I and type III IFN signaling we refined model components of the JAK-STAT signaling pathway available in the literature [S27, S28, S29, S30, S31]. Primarily, based on the available models we have listed variables corresponding to the major biochemical species involved in signaling. The list of model variables, together with initial values used in simulations, is presented in Supplementary Table 1.

Initial values of the copy number of STAT1 and STAT2 proteins as well as both receptors complexes respectively, were sampled as described in Section 3.4.

Phosphorylated STAT molecules reside in nucleus and are dephosphorylated in a stepwise process. Dephosphorylation introduces a delay of nuclear export of phosphorylated STAT dimers [S32], which in the model is represented by a chain of linear reactions [S27, S33, S34]. The number of STAT1/1 and STAT1/2 dimers in the nucleus in the intermediate states are denotes as $Y_{1/1}^{(i)}$ and $Y_{1/2}^{(i)}$, respectively, for i changing form 1 to 5. The total copy number of STAT1/1 and STAT1/2 in the nucleus is the sum of intermediate, partly dephosphorylated forms

$$Y_{1/1} = \sum_{i=1}^{5} Y_{1/1}^{(i)}, \quad Y_{1/2} = \sum_{i=1}^{5} Y_{1/2}^{(i)}.$$

variable	init. cond.	unit	description	Reference
Y_1	$\mu_1 = 1000$	[molecules]	inactive type I receptor	[S19]
Y_2	0	[molecules]	active type I receptor	
Y_3	$\mu_3 = 1000$	[molecules]	inactive type III receptor	[S19]
Y_4	0	[molecules]	active type III receptor	
Y_5	$\mu_5 = 1.659 \cdot 10^5$	[molecules]	STAT 1 in the cytoplasm	[S33, S35]
Y_6	$\mu_6 = 1.659 \cdot 10^5$	[molecules]	STAT 2 in the cytoplasm	[S33, S35]
Y_7	0	[molecules]	phosphorylated STAT 1 in the cytoplasm	Assumed
Y_8	0	[molecules]	phosphorylated STAT 2 in the cytoplasm	Assumed
Y_9	0	[molecules]	p-STAT 1/1 in the cytoplasm	Assumed
Y_{10}	0	[molecules]	p-STAT 1/2 in the cytoplasm	Assumed
$Y_{1/1}^{(i)}$	0	[molecules]	i-the intermediate form of p-STAT 1/1 in the nucleus ($i = 1, ..., 5$)	Assumed
$Y_{1/2}^{(i)}$	0	[molecules]	i-the intermediate form of p-STAT 1/2 in the nucleus ($i = 1, ..., 5$)	Assumed
$Y_{1/1}$	0	[molecules]	total p-STAT 1/1 in the nucleus ($Y_{1/1} = \sum_{i=1}^{5} Y_{1/1}^{(i)}$)	Assumed
$Y_{1/2}$	0	[molecules]	total p-STAT 1/2 in the nucleus ($Y_{1/2} = \sum_{i=1}^{5} Y_{1/2}^{(i)}$)	Assumed
x_α	0-5	[ng/ml]	concentration of IFN-α	[S36, S37]
$x_{\lambda 1}$	0-250	[ng/ml]	concentration of IFN-λ1	[S36, S37]

Supplementary Table 1: Variables of the IFN signaling model and their initial conditions. The initial copy number of STAT1 and STAT2 proteins as well as both receptors was assumed to be random therefore their means, μ_i, are presented.

3.2 Reactions and their rates

Similarly to the model variables, we have identified major reactions involved in type I and type III IFN signaling based on the existing models [S27, S28, S29, S30, S31]. Below we list all model reactions together with their rates. Reaction rates were formulated using mass action kinetics. Each arrow represents a single reaction with substrates to the left, products to the right and rate over the arrow.

Receptor binding/unbinding

(Type I receptor – IFN-α binding) $Y_1 \xrightarrow{x_\alpha k^+_{R_I} Y_1} Y_2$

(Type I receptor – IFN-α unbinding) $Y_2 \xrightarrow{k^-_{R_I} Y_2} Y_1$

(Type III receptor – IFN-λ1 binding) $Y_3 \xrightarrow{x_{\lambda 1} k^+_{R_{III}} Y_3} Y_4$

(Type III receptor – IFN-λ1 unbinding) $Y_4 \xrightarrow{k^-_{R_{III}} Y_4} Y_3$

STATs phosphorylation

(STAT1 phosphorylation) $Y_5 \xrightarrow{Y_2 k^{R_I}_{S1} Y_5} Y_7$

$Y_5 \xrightarrow{Y_4 k^{R_{III}}_{S1} Y_5} Y_7$

(STAT2 phosphorylation) $Y_6 \xrightarrow{Y_2 k^{R_I}_{S2} Y_6} Y_8$

$Y_6 \xrightarrow{Y_4 k^{R_{III}}_{S2} Y_6} Y_8$

phosphorylated STATs (pSTATs) dimerisation

(dimerisation p-STAT1/1) $\begin{matrix} Y_7 \\ Y_7 \end{matrix} \xrightarrow{k_{S1S1} Y_7^2} Y_9$

(dimerisation p-STAT1/2) $\begin{matrix} Y_7 \\ Y_8 \end{matrix} \xrightarrow{k_{S1S2} Y_7 Y_8} Y_{10}$

Homo- and heterodimers in the nucleus

(p-STAT1/1 cytoplasm - nucleus translocation) $Y_9 \xrightarrow{k_{N11} Y_9} Y^{(1)}_{1/1}$

(p-STAT1/2 cytoplasm - nucleus translocation) $Y_{10} \xrightarrow{k_{N12} Y_{10}} Y^{(1)}_{1/2}$

(p-STAT1/1 dephosphorylation) $Y^{(1)}_{1/1} \xrightarrow{k_{N11} Y^{(1)}_{1/1}} Y^{(2)}_{1/1} \dots Y^{(4)}_{1/1} \xrightarrow{k_{N11} Y^{(4)}_{1/1}} Y^{(5)}_{1/1}$

(p-STAT1/2 dephosphorylation) $Y^{(1)}_{1/2} \xrightarrow{k_{N12} Y^{(1)}_{1/2}} Y^{(2)}_{1/2} \dots Y^{(4)}_{1/2} \xrightarrow{k_{N12} Y^{(4)}_{1/2}} Y^{(5)}_{1/2}$

(p-STAT1/1 nucleus - cytoplasm translocation) $Y^{(5)}_{1/1} \xrightarrow{k_{C11} Y^{(5)}_{1/1}} \begin{matrix} Y_5 \\ Y_5 \end{matrix}$

(p-STAT1/2 nucleus - cytoplasm translocation) $Y^{(5)}_{1/2} \xrightarrow{k_{C12} Y^{(5)}_{1/2}} \begin{matrix} Y_5 \\ Y_6 \end{matrix}$

3.3 Model parameters

Parameter values used in the simulations are presented in Supplementary Table 2. The majority of the parameter values has been adapted from [S33, S35] and expressed in units corresponding to copy numbers instead of concentrations assuming cytoplasmic and nuclear volume as in [S33, S35].

Parameters of receptor activation and deactivation were varied to examine possible, and currently unknown, differences in kinetics of these receptors. Specifically, the rate of deactivation of type I receptors, $k^-_{R_I}$, was set, to reflect the time-frame of nuclear phosphorylated STATs activity reported in the experimental data of [S37]. The rate of deactivation of type III receptors, was then dictated by the coeffecient $\delta = \frac{k^-_{R_{III}}}{k^-_{R_I}}$. Furthermore, values of receptors' K_d's, $K^I_d = \frac{k^-_{R_I}}{k^+_{R_I}}$ and $K^{III}_d = \frac{k^-_{R_{III}}}{k^+_{R_{III}}}$ has been set to correspond to experimentally observed data [S37]. In consequence, rates of receptors' activation $k^+_{R_I}$ and $k^+_{R_{III}}$, were determined by both K_d's and deactivation rates $k^-_{R_I}$ and $k^-_{R_{III}}$.

parameter	value	unit	description	Reference
$k^-_{R_I}$	0.78	$[\frac{1}{\text{min.}}]$	ligand unbinding rate of type I receptor	Assumed, [S37]
$k^-_{R_{III}}$	variable	$[\frac{1}{\text{min.}}]$	ligand unbinding rate of type III receptor	Assumed
$k^+_{R_I}$	variable	$[\frac{1}{\text{ng/ml}\cdot\text{min.}}]$	ligand binding rate of type I receptor	Assumed
$k^+_{R_{III}}$	variable	$[\frac{1}{\text{ng/ml}\cdot\text{min.}}]$	ligand binding rate of type III receptor	Assumed
$K^I_d = k^-_{R_I}/k^+_{R_I}$	0.5	$[\frac{\text{ng}}{\text{ml}}]$	dissociation constant of the type I receptor	[S19, S38]
$K^{III}_d = k^-_{R_{III}}/k^+_{R_{III}}$	25	$[\frac{\text{ng}}{\text{ml}}]$	dissociation constant of the type III receptor	[S19, S38]
$k^{R_I}_{S1}$	0.0202	$[\frac{1}{\text{molecules}\cdot\text{min.}}]$	STAT1 phosphorylation rate by type I receptor	[S33, S35]
$k^{R_{III}}_{S1}$	0.0202	$[\frac{1}{\text{molecules}\cdot\text{min.}}]$	STAT1 phosphorylation rate by type III receptor	[S33, S35]
$k^{R_I}_{S2}$	0.0202	$[\frac{1}{\text{molecules}\cdot\text{min.}}]$	STAT2 phosphorylation rate by type I receptor	[S33, S35]
$k^{R_{III}}_{S2}$	0.0202	$[\frac{1}{\text{molecules}\cdot\text{min.}}]$	STAT2 phosphorylation rate by type III receptor	[S33, S35]
k_{S1S1}	0.001362	$[\frac{1}{\text{molecules}\cdot\text{min.}}]$	p-STAT1/1 dimerisation rate	[S33, S35]
k_{S1S2}	0.001362	$[\frac{1}{\text{molecules}\cdot\text{min.}}]$	p-STAT1/2 dimerisation rate	[S33, S35]
$k_{N1/1}$	0.315537	$[\frac{1}{\text{min.}}]$	p-STAT1/1 nuclear translocation rate	[S33, S35]
$k_{1/1}$	2.640399	$[\frac{1}{\text{min.}}]$	p-STAT1/1 nuclear dephosphorylation rate	[S33, S35]
$k_{C1/1}$	2.640399	$[\frac{1}{\text{min.}}]$	p-STAT1/1 cytoplasmic translocation rate	[S33, S35]
$k_{N1/2}$	0.315537	$[\frac{1}{\text{min.}}]$	p-STAT1/2 nuclear translocation rate	[S33, S35]
$k_{1/2}$	2.640399	$[\frac{1}{\text{min.}}]$	p-STAT1/2 nuclear dephosphorylation rate	[S33, S35]
$k_{C1/2}$	2.640399	$[\frac{1}{\text{min.}}]$	p-STAT1/2 cytoplasmic translocation rate	[S33, S35]

Supplementary Table 2: Parameters used in simulations of the IFN signaling model. Parameters of receptor activation and deactivation were being varied to examine the impact of differences in kinetics of these receptors on information capacity of the pathway.

3.4 Modeling stochasticity

We have assumed that the stochasticity of the model arises from two sources: (i) stochasticity of all individual reactions, and (ii) variability of total copy numbers of signaling proteins. Stochasticity of individual reactions was modeled according to the Chemical Master Equation [S39] and for numerical computations approximated by the Linear Noise Approximation within the StochSens Matlab Package [S40]. Extrinsic noise was introduced as the variation of total copy number of STAT1 and STAT2 proteins as well as both receptors complexes. Specifically, we assumed that the initial values of variables Y_1, Y_3, Y_5, Y_6, which correspond to the copy number of STAT1 and STAT2 proteins as well as both receptors complexes respectively, were random, with mean μ_i and standard deviation σ_i for $i \in \{STAT1,\ STAT2,\ R_I,\ R_{III}\}$. The framework of the StochSens package, similarly to the

fluctuation-dissipation theorem, approximates any distribution of the initial copy number with the normal distribution with unchanged mean and standard deviation. We assumed that the standard deviations σ_i's, relate to the means μ_i's, through coefficient of variation,

$$\sigma_i = c_v \ \mu_i.$$

The coefficient of variation was varied as described in the main paper, whereas the means, μ_i's, are give in Supplementary Table 1.

3.5 Input and output

Calculation of the information capacity requires specification of the input and output. We have defined the input-output relationship of the IFN signaling system as

$$\begin{array}{ccc} \text{(Input)} & & \text{(Output)} \\ x = (x_\alpha, x_{\lambda 1}) & \longrightarrow & Y = \big(Y_{1/2}(t_1), \ldots, Y_{1/2}(t_n), Y_{1/1}(t_1), \ldots, Y_{1/1}(t_n)\big) \end{array},$$

where the input concentrations $x = (x_\alpha, x_{\lambda 1})$ define concentrations of a 30-minutes stimulation pulse followed by the washout. Precisely, the concentrations of both IFNs over time, denoted as $x(t) = (x_\alpha(t), x_{\lambda 1}(t))$ relate to the input values $x = (x_\alpha, x_{\lambda 1})$ in the following way

$$x(t) = \begin{cases} (x_\alpha,\ x_{\lambda 1}) \ \left[\frac{\text{ng}}{\text{ml}}\right] & \text{for} \quad t \in [0, 30] \\ (0,0) \ \left[\frac{\text{ng}}{\text{ml}}\right] & \text{for} \quad t > 30 \end{cases}.$$

We considered x_α and $x_{\lambda 1}$ to vary from 0 to 5 and 250 ng/ml, respectively. The maximal values have been selected to represent saturating doses according to [S37].

3.6 Numerical computations

The specification of the model described above allowed us to simulate model trajectories (Fig. 3 of the main paper, Supplementary Figure 4 and Supplementary Figure 5) as well as to calculate the Fisher information. Calculations were performed using the methodology described in [S41], which is based on the Linear Noise Approximation of the Chemical Master Equation, and is implemented as the Matlab package StochSens [S40]. In order to calculate C_A^* according to the Eq. S.9 we simulated the model for each point from a mesh composed of different combinations of IFNs concentrations, calculated FIMs at those points, and evaluated the integral given in Eq. S.9.

3.7 Optimal input distributions

The channel capacity, C_A^*, of IFN signaling is shown in the Fig. 4 of the main paper. The corresponding two-dimensional optimal input distributions, $P_{JP}^*(X)$, are depicted in Supplementary Figure 6 as heatmaps.

3.7.1 Singular FIMs

Calculation of the information capacity C_A^* according to the Eq. S.9 for multidimensional inputs requires calculation of the determinant of FIMs. It is known that if a matrix is singular, the calculation of its determinant can be numerically unstable. Therefore, to ensure accuracy of computations, we considered FIM(x) to be singular a if its (L_1-norm) condition number is larger than 10^3 and set $|\text{FIM}(X)| = 0$ for these values of x. L_1-norm condition number of a matrix $A = \{a_{ij}\}$ is defined as $\text{cond}(A) = \|A\| \cdot \|A^{-1}\|$, where $\|A\| = \max_j \sum_i |a_{ij}|$.

3.8 Joint capacity of N cells, C_N^*

As described in the main paper, our framework allows quantifying differences between information transfer at the single cell and population levels.
Precisely, Eq. 8 of the main paper, describes the joint capacity of the N cells as the sum of the asymptotic capacity, C_A^*, and the factor dependent on the number of cells, $1/2\log_2(N)$. Therefore, as long as $\int_{\mathcal{X}} |FIM(x)|^{\frac{1}{2}} dx > 0$, or equivalently $C_A^* > -\infty$, the capacity of N cells, C_N^* increases with N. The same equation, as well as our numerical simulations of the *Validity* section, suggests that the capacity of a single cell could be roughly approximated by C_A^* alone.
As shown in Fig. 4 of the main paper, in the presence of high noise and similar receptor kinetics, C_{A}^* reaches negative values, e.g. for $\delta = 0.9$ and $c_v = 1.5$, $C_{\mathrm{A}}^* \approx -1$. Obviously, in this case C_A^* is not a good approximation of C_1^*, as $C_N^* > 0$ for all N. However, $C_{\mathrm{A}}^* < 0$ is indicative of low value of the capacity of the single cell C_1^*, presumable lower than 2 bits.
In addition to the above heuristic interpretation, C_{A}^* dictates the level of C_N^*, for large N through Eq. 8 of the main paper. This is illustrated in Supplementary Figure 7A that shows capacities C_N^* as a function of N for $\delta = 0.9$ and $c_v = 1.5$. The above argument demonstrates that even if the capacity of a single cell is insufficient to distinguish the presence and absence of both IFNs, for a sufficiently large population size a necessary discriminatory power can be obtained. In Supplementary Figure 7B we show capacities C_N^* for the highest considered similarity of the IFN receptors $\delta = 0.99$ and all considered noise levels. This further demonstrates that even with minor kinetic differences and high noise levels, the capacity of cellular populations can be high.

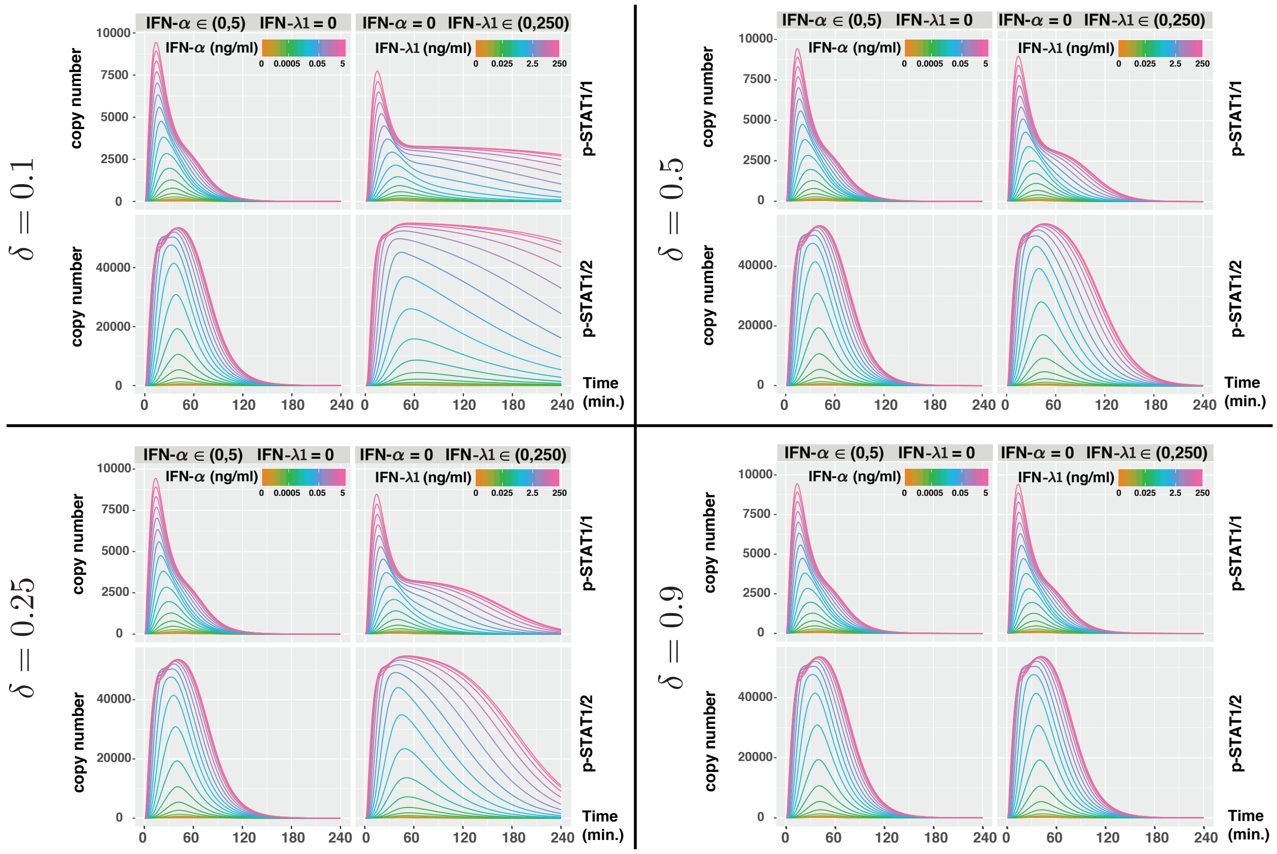

Supplementary Figure 4: Average trajectories of the copy number of nuclear p-STAT1/1 ($Y_{1/1}$) and of nuclear p-STAT1/2 ($Y_{1/2}$) upon 30 minutes stimulation of either IFN-α or IFN-λ1. Panels correspond to different values of the differential kinetics coefficient, δ. Colours denote doses of IFN-α or IFNλ1. In the LNA framework used for simulations, average trajectories are independent on the value of the coefficient of variation, c_v.

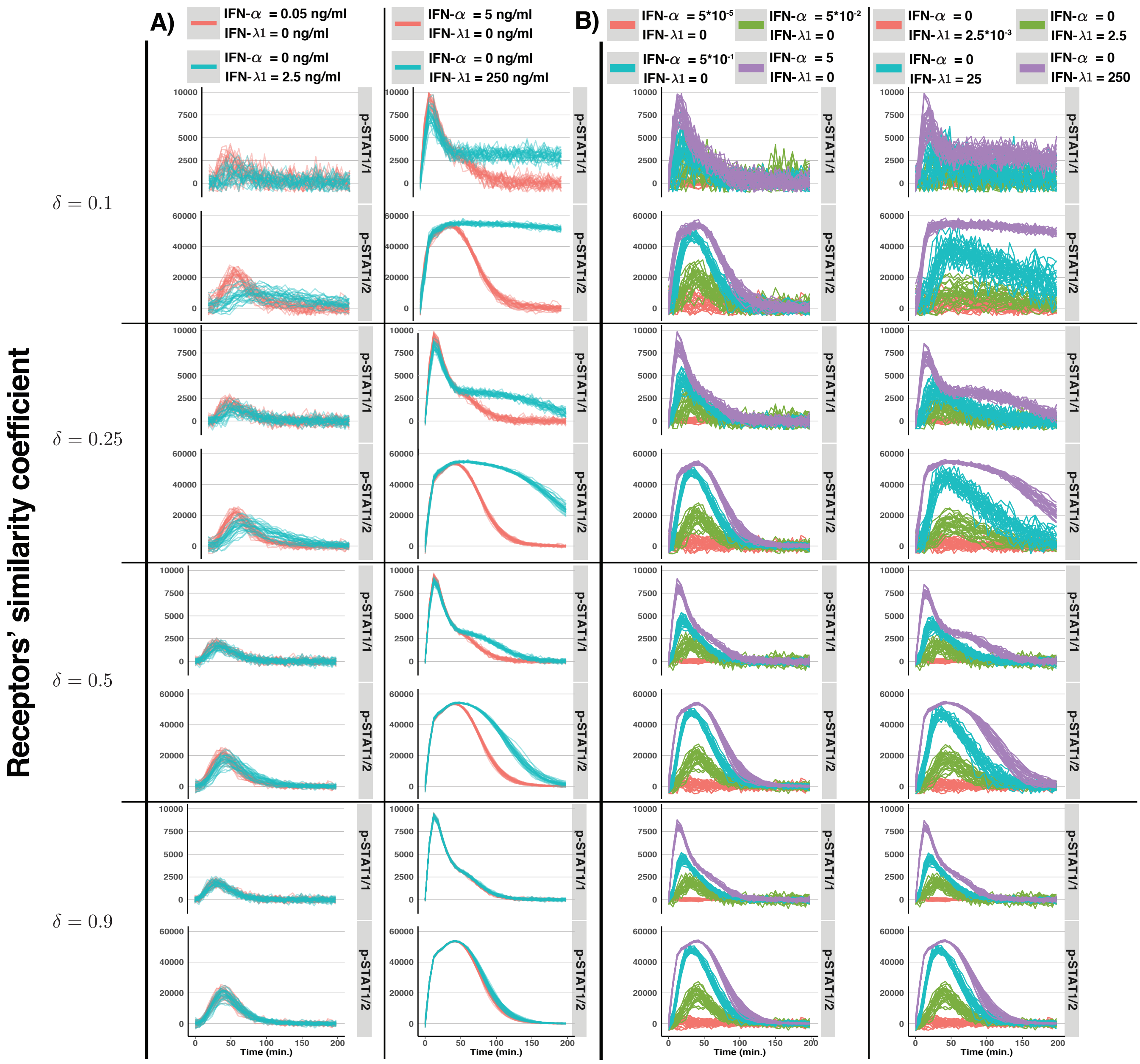

Supplementary Figure 5: Trajectories of the copy number of nuclear p-STAT1/1 ($Y_{1/1}$) and of nuclear p-STAT1/2 ($Y_{1/2}$) upon 30 minutes stimulation with an indicated concentration of IFN-α and IFN-λ1. **(A)** Comparison of responses to stimulation with intermediate (left column) and high (right column) doses both IFNs. **(B)** Comparison of responses to increasing doses of IFN-α (left column) and IFN-λ1 (right) column. Rows of each both panel correspond to different values of the differential kinetics coefficient, δ. To ensure that trajectories plotted for different parameter values can be distinguished we used $c_v = 0$ for all simulations. Each panel contains 30 trajectories.

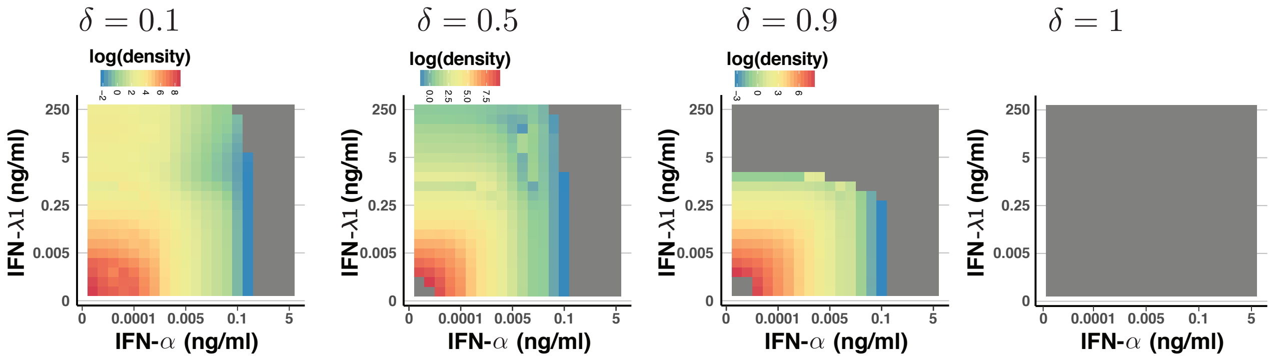

Supplementary Figure 6: Optimal input distributions, $P^*_{JP}(X_\alpha, X_{\lambda 1})$, of IFN signaling model for different values of the differential kinetics coefficient, δ, with the coeffecient of vatiotion $c_v = 0$. Horizontal and vertical axes as well as the value of density are in the log-scale. Gray colour indicates singularity of FIMs.

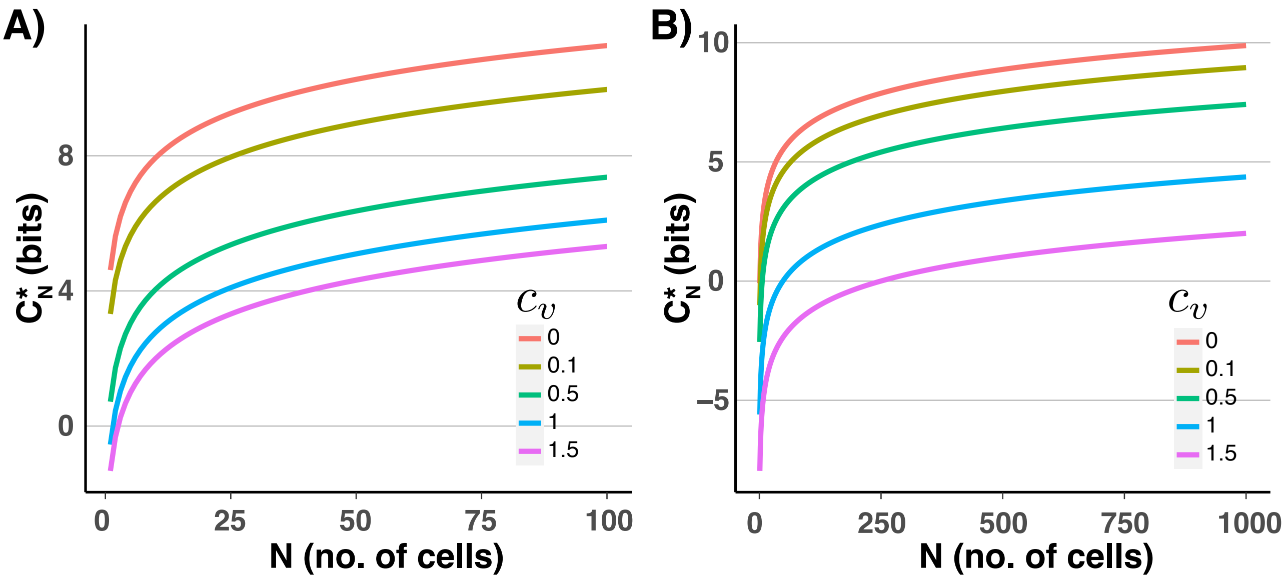

Supplementary Figure 7: The capacity of population of N, C^*_N, given by Eq. S.8 for $\delta = 0.9$ (**A**) and $\delta = 0.99$ (**B**) and different values of the coefficient of variation, c_v. The plot corresponds to Fig. 4C of the main paper.

4 Methods to calculate the Fisher Information

The FIM is defined as the average product of gradients of log-density, Eq. S.8. Calculation of the FIM is crucial for computation of the asymptotic capacity, Eq. S.9. Therefore, below, we briefly discuss available approaches to calculate FIM. Broadly speaking, these can be divided into three groups.

1. *Using an explicit-formula.* For several distributions an explicit formula describing FIM is available. Precisely, for simple distributions, $P(Y|X = x)$, specifically one-dimensional, e.g. normal, log-normal, exponential, etc., Eq. S.6 yields analytical expressions, which can be used to evaluate Eq. S.9. Explicit formulae are also available in few multivariate scenarios [S42]. Linear noise approximation, used to calculate FIMs of the interferon signaling model, leads to the multivariate normal approximation of the output distribution, for which an explicit formula for the FIM is available [S41].

2. *Numerical evaluation of Eq. S.8.* Numerical computation of log-density derivatives (or Hessian matrix) and numerical integration of these log-densities provides a natural recipe for calculating FIM. Our test example (biochemical sensor model) uses this approach. This method, however, can be computationally problematic for high-dimensional distributions. Several computational approaches are available to improve efficiency and accuracy of computations for high number of dimensions (>10) [S43, S44, S45]. In addition, recently, a method to calculate the Fisher information based on finite state projection has been proposed in [S46].

3. *Non-parametric estimation.* Since the FIM depends directly on the probability density function, its estimation can be achieved through density estimation [S47]. A good estimate of FIM, however, requires a good estimate of the density as well as of the derivative of log-density. In addition to conventional kernel density estimators, alternative approaches were tested, e.g., [S48] found a unique interpolation of the cumulative distribution function that maximizes the FIM, while [S49] adapted the maximized penalized likelihood method of [S50]. Other interesting directions, including for multivariate scenarios, are discussed in [S49, S51, S52]. Besides, recently, novel methods that calculate FIM directly from the data in an efficient way have been proposed [S53, S54]. Nonetheless, the performance of the first one was shown only in 1- and 2-dimensional case, while the other was tested in an elementary setting. Still, it would be interesting to merge these methods with our approach to calculate channel capacity via Jeffrey's prior rule.

In systems biology, stochastic models are often described by Chemical Master Equation and simulated using Gillespie algorithm [S55]. Then FIM can be then obtained using extensive Monte Carlo algorithms [S56, S57]. Here, we simulated the interferon signaling model using the LNA approximation. For LNA, a closed-formula for the FIM exists and enables efficient computations as described in [S41] and implemented as the Matlab package in [S40].

5 Computational efficiency of the algorithm

The algorithm to calculate the asymptotic capacity, C_A^*, Eq. S.9, can be divided into four main steps: 1) construction of an input values grid ; 2) calculation FIM for each input value on the grid; 3) integration according to the Eq. S.9. Therefore, its computational cost depends on three factors:

1. dimension of the input,

2. cost of calculating the FIM for each input value on the input grid,

3. cost of calculating the integral of Eq. S.9,

Number of time points	Time of computations
1	13 minutes
4	64 minutes
10	123 minutes
20	189 minutes

Supplementary Table 3: Computational time to obtain capacities presented in Figure 4A of the main paper as function of the length of the output.

The computational cost can be simplistically represented as

$$q^{d_x} \cdot (C_1 + C_2)\,,$$

where q is the number of points in each dimension of the input grid, d_x is the dimension of the input, C_1 is the cost of calculating FIM for each point on the input grid and C_2 is the point cost of integration Eq. S.9. Therefore, the computational cost is exponentially dependent on the dimension of the input. However, the cost of calculating FIMs, itself, depends on the dimension of the input. Moreover, the cost of calculating the FIM also depends on the dimension of the output and model complexity, i.e., the number of modelled variables, non-linearities, timescales, etc.. Given that cost of calculating FIM depends on the specific method (see the previous section), it is hard to provide a general cost calculation. Therefore, we provide practical computational times for the interferon signaling model. The interferon signaling model involved over 20 biochemical species, 2-dimensional input, and up to 40-dimensional output. For the model, we measured computation times on a workstation with Intel® Xeon® CPU E5-1650 v3 3.5GHz, 6 cores (12 logical units), 32 GB RAM DDR4 2133 MHz (Matlab's Parallel Computing Toolbox was used in simulations with 4 cores). Computation times for various model setting of Figure 4A are reported in Supplementary Table 3.

6 Extensions to estimate information capacity from experimental data

Our framework aims at quantification of information capacity using models of signaling pathways given by a probability distribution $P(Y|X = x)$. The proposed approach could, however, be applied in any scenario, in which the Fisher information is available. If experimental measurements provided sufficient data to estimate Fisher information, the information capacity C^*_A could be calculated within our framework. This results from the fact that the Fisher information is enough to evaluate the integral in Eq. S.9. Several methods have been recently proposed to estimate Fisher information directly from experimental measurements (e.g. [S53, S54]). Hence the proposed methods could be further utilised to quantify information capacity from experimental data.

Supplementary References

[S1] Richard E Blahut. Computation of channel capacity and rate-distortion functions. *IEEE Transactions on Information Theory*, 18(4):460–473, 1972.

[S2] Thomas M Cover and Joy A Thomas. *Elements of Information Theory.* John Wiley & Sons, 2012.

[S3] Gašper Tkačik, Curtis G Callan Jr, and William Bialek. Information capacity of genetic regulatory elements. *Physical Review E*, 78(1):011910, 2008.

[S4] Raymond Cheong, Alex Rhee, Chiaochun Joanne Wang, Ilya Nemenman, and Andre Levchenko. Information transduction capacity of noisy biochemical signaling networks. *Science*, 334(6054): 354–358, 2011.

[S5] Susanne Schreiber, Christian K Machens, Andreas VM Herz, and Simon B Laughlin. Energy-efficient coding with discrete stochastic events. *Neural Computation*, 14(6):1323–1346, 2002.

[S6] Vijay Balasubramanian, Don Kimber, and Michael J Berry II. Metabolically efficient information processing. *Neural Computation*, 13(4):799–815, 2001.

[S7] Frédéric Dupuis, Wei Yu, and F Willems. Blahut-arimoto algorithms for computing channel capacity and rate-distortion with side information. In *IEEE International Symposium on Information Theory*, pages 179–179, 2004.

[S8] Jose M Bernardo. Reference posterior distributions for bayesian inference. *Journal of the Royal Statistical Society. Series B (Methodological)*, pages 113–147, 1979.

[S9] BS Clarke and AR Barron. Jeffreys' prior is asymptotically least favorable under entropy risk. *Journal of Statistical Planning and Inference*, 1994.

[S10] James O Berger, José M Bernardo, and Dongchu Sun. The formal definition of reference priors. *The Annals of Statistics*, pages 905–938, 2009.

[S11] AM Walker. On the asymptotic behaviour of posterior distributions. *Journal of the Royal Statistical Society. Series B (Methodological)*, pages 80–88, 1969.

[S12] Harold Jeffreys. An invariant form for the prior probability in estimation problems. *Proceedings of the Royal Society of London. Series A. Mathematical and Physical Sciences*, 186(1007):453–461, 1946.

[S13] J.M. Bernardo and A.F.M. Smith. Bayesian theory. *Measurement Science and Technology*, 12: 221–222, 2001.

[S14] James O Berger and José M Bernardo. On the development of reference priors. *Bayesian Statistics*, 4(4):35–60, 1992.

[S15] Nicolas Brunel and Jean-Pierre Nadal. Mutual information, fisher information, and population coding. *Neural Computation*, 10(7):1731–1757, 1998.

[S16] Lucien Le Cam. *Asymptotic Methods in Statistical Decision Theory.* Springer Science & Business Media, 2012.

[S17] Abraham Wald. Tests of statistical hypotheses concerning several parameters when the number of observations is large. *Transactions of the American Mathematical Society*, 54(3):426–482, 1943.

[S18] Thomas R O'Brien, Ludmila Prokunina-Olsson, and Raymond P Donnelly. IFN-λ4: the paradoxical new member of the interferon lambda family. *Journal of Interferon & Cytokine Research*, 34(11):829–838, 2014.

[S19] Stephan Wilmes, Oliver Beutel, Zhi Li, Véronique Francois-Newton, Christian P Richter, Dennis Janning, Cindy Kroll, Patrizia Hanhart, Katharina Hötte, Changjiang You, et al. Receptor dimerization dynamics as a regulatory valve for plasticity of type I interferon signaling. *Journal of Cell Biology*, 209(4):579–593, 2015.

[S20] Nicole A De Weerd, Julian P Vivian, Thao K Nguyen, Niamh E Mangan, Jodee A Gould, Susie-Jane Braniff, Leyla Zaker-Tabrizi, Ka Yee Fung, Samuel C Forster, Travis Beddoe, et al. Structural basis of a unique interferon-β signaling axis mediated via the receptor IFNAR1. *Nature Immunology*, 14(9):901–907, 2013.

[S21] Gideon Schreiber and Jacob Piehler. The molecular basis for functional plasticity in type I interferon signaling. *Trends in Immunology*, 36(3):139–149, 2015.

[S22] George R Stark and James E Darnell. The JAK-STAT pathway at twenty. *Immunity*, 36(4): 503–514, 2012.

[S23] Andreas Wack, Ewa Terczyńska-Dyla, and Rune Hartmann. Guarding the frontiers: the biology of type III interferons. *Nature Immunology*, 16(8):802–809, 2015.

[S24] Adrian Egli, Deanna M Santer, Daire O'shea, D Lorne Tyrrell, and Michael Houghton. The impact of the interferon-lambda family on the innate and adaptive immune response to viral infections. *Emerging Microbes & Infections*, 3(7):e51, 2014.

[S25] Christopher R Bolen, Siyuan Ding, Michael D Robek, and Steven H Kleinstein. Dynamic expression profiling of type I and type III interferon-stimulated hepatocytes reveals a stable hierarchy of gene expression. *Hepatology*, 59(4):1262–1272, 2014.

[S26] Nikolaus Jilg, Wenyu Lin, Jian Hong, Esperance A Schaefer, David Wolski, James Meixong, Kaku Goto, Cynthia Brisac, Pattranuch Chusri, Dahlene N Fusco, et al. Kinetic differences in the induction of interferon stimulated genes by interferon-α and interleukin 28B are altered by infection with hepatitis C virus. *Hepatology*, 59(4):1250–1261, 2014.

[S27] J. Vanlier, CA Tiemann, PAJ Hilbers, and NAW van Riel. An integrated strategy for prediction uncertainty analysis. *Bioinformatics*, 2012.

[S28] Mikołaj Rybiński and Anna Gambin. Model-based selection of the robust JAK-STAT activation mechanism. *Journal of Theoretical Biology*, 309:34–46, 2012.

[S29] Anna Gambin, Agata Charzyńska, Aleksandra Ellert-Miklaszewska, and Mikołaj Rybiński. Computational models of the JAK1/2-STAT1 signaling. *Jak-Stat*, 2(3):e24672, 2013.

[S30] Jaroslaw Smieja, Mohammad Jamaluddin, Allan R Brasier, and Marek Kimmel. Model-based analysis of interferon-β induced signaling pathway. *Bioinformatics*, 24(20):2363–2369, 2008.

[S31] Tomasz Jetka, Agata Charzyńska, Anna Gambin, Michael PH Stumpf, and Michał Komorowski. StochDecomp Matlab package for noise decomposition in stochastic biochemical systems. *Bioinformatics*, 30(1):137–138, 2014.

[S32] N MacDonald. Time delay in simple chemostat models. *Biotechnology and Bioengineering*, 18 (6):805–812, 1976.

[S33] I Swameye, T G Müller, J. Timmer, O Sandra, and U. Klingmüller. Identification of nucleocytoplasmic cycling as a remote sensor in cellular signaling by databased modeling. *Proceedings of the National Academy of Sciences*, 100(3):1028–1033, January 2003. doi: 10.1073/pnas.0237333100. URL `http://www.pnas.org/cgi/doi/10.1073/pnas.0237333100`.

[S34] Andreas Raue, Clemens Kreutz, Thomas Maiwald, Julie Bachmann, Marcel Schilling, Ursula Klingmüller, and Jens Timmer. Structural and practical identifiability analysis of partially observed dynamical models by exploiting the profile likelihood. *Bioinformatics*, 25(15):1923–1929, 2009.

[S35] J. Vanlier, CA Tiemann, PAJ Hilbers, and NAW van Riel. A bayesian approach to targeted experiment design. *Bioinformatics*, 2012.

[S36] Zuzanna Makowska, Francois HT Duong, Gaia Trincucci, David F Tough, and Markus H Heim. Interferon-β and interferon-λ signaling is not affected by interferon-induced refractoriness to interferon-α in vivo. *Hepatology*, 53(4):1171–1180, 2011.

[S37] Stephen G Maher, Faruk Sheikh, Anthony J Scarzello, Ana L Romero-Weaver, Darren P Baker, Raymond P Donnelly, and Ana M Gamero. IFN-α and IFN-λ differ in their antiproliferative effects and duration of JAK/STAT signaling activity. *Cancer biology & therapy*, 7(7):1109–1115, 2008.

[S38] Martynas Gavutis, Suman Lata, Peter Lamken, Pia Müller, and Jacob Piehler. Lateral ligand-receptor interactions on membranes probed by simultaneous fluorescence-interference detection. *Biophysical Journal*, 88(6):4289–4302, 2005.

[S39] N.G. Van Kampen. *Stochastic Processes in Physics and Chemistry.* North Holland, 2006.

[S40] Michał Komorowski, Justina Žurauskienė, and Michael PH Stumpf. StochSens–matlab package for sensitivity analysis of stochastic chemical systems. *Bioinformatics*, 28(5):731–733, 2012.

[S41] Michal Komorowski, Maria J Costa, David A Rand, and Michael P H Stumpf. Sensitivity, robustness, and identifiability in stochastic chemical kinetics models. *Proceedings of the National Academy of Sciences*, 108(21):8645–8650, May 2011. doi: 10.1073/pnas.1015814108.

[S42] Louis L Scharf. *Statistical signal processing*, volume 98. Addison-Wesley Publishing, 1991.

[S43] James C Spall. Monte carlo computation of the fisher information matrix in nonstandard settings. *Journal of Computational and Graphical Statistics*, 14(4):889–909, 2005.

[S44] Sonjoy Das, James C Spall, and Roger Ghanem. Efficient monte carlo computation of fisher information matrix using prior information. *Computational Statistics & Data Analysis*, 54(2): 272–289, 2010.

[S45] Miroslav Šimandl, Jakub Královec, and Petr Tichavskỳ. Filtering, predictive, and smoothing cramér–rao bounds for discrete-time nonlinear dynamic systems. *Automatica*, 37(11):1703–1716, 2001.

[S46] Zachary R Fox and Brian Munsky. The finite state projection based fisher information matrix approach to estimate and maximize the information in single-cell experiments. *bioRxiv*, page 370205, 2018.

[S47] Bernard W Silverman. *Density estimation for statistics and data analysis.* Routledge, 2018.

[S48] Peter J Huber. Fisher information and spline interpolation. *The Annals of Statistics*, pages 1029–1033, 1974.

[S49] Lubomir Kostal and Ondrej Pokora. Nonparametric estimation of information-based measures of statistical dispersion. *Entropy*, 14(7):1221–1233, 2012.

[S50] IJ Goodd and Ray A Gaskins. Nonparametric roughness penalties for probability densities. *Biometrika*, 58(2):255–277, 1971.

[S51] Hiroaki Sasaki, Yung-Kyun Noh, Gang Niu, and Masashi Sugiyama. Direct density derivative estimation. *Neural computation*, 28(6):1101–1140, 2016.

[S52] José E Chacón, Tarn Duong, and MP Wand. Asymptotics for general multivariate kernel density derivative estimators. *Statistica Sinica*, pages 807–840, 2011.

[S53] Visar Berisha and Alfred O Hero. Empirical non-parametric estimation of the fisher information. *IEEE Signal Processing Letters*, 22(7):988–992, 2015.

[S54] Omri Har-Shemesh, Rick Quax, Borja Miñano, Alfons G Hoekstra, and Peter MA Sloot. Nonparametric estimation of fisher information from real data. *Physical Review E*, 93(2):023301, 2016.

[S55] Daniel T Gillespie. Exact stochastic simulation of coupled chemical reactions. *The journal of physical chemistry*, 81(25):2340–2361, 1977.

[S56] R. Gunawan, Y. Cao, L. Petzold, and F.J. Doyle III. Sensitivity analysis of discrete stochastic systems. *Biophysical Journal*, 88(4):2530–2540, 2005.

[S57] M. Rathinam, P.W. Sheppard, and M. Khammash. Efficient computation of parameter sensitivities of discrete stochastic chemical reaction networks. *The Journal of Chemical Physics*, 132: 034103, 2010.
